# *TAS2R38* taste receptor gene and chronic rhinosinusitis: new data from an Italian population

**DOI:** 10.1186/s12881-016-0321-3

**Published:** 2016-08-11

**Authors:** Stefania Gallo, Sarah Grossi, Giulia Montrasio, Giorgio Binelli, Raffaella Cinquetti, Daniel Simmen, Paolo Castelnuovo, Paola Campomenosi

**Affiliations:** 1 Clinica Otorinolaringoiatrica, Ospedale di Circolo e Fondazione Macchi, Università degli Studi dell’Insubria, Varese, Italy; 2 Dipartimento di Biotecnologie e Scienze della Vita (DBSV), Università dell’Insubria, Via J.H. Dunant, 3, Varese, 21100 Italy; 3 Center for Rhinology, Skull Base Surgery and Facial Plastic Surgery, ORL-Zentrum, Klinik Hirslanden, Zurich, Switzerland; 4 The Protein Factory, Centro Interuniversitario di Ricerca in Biotecnologie Proteiche, Politecnico di Milano, ICRM-CNR Milano and Università degli Studi dell’Insubria, Varese, Italy

**Keywords:** Bitter taste receptor T2R38, Chronic rhinosinusitis, Pathogenesis, Genotyping, *TAS2R38* gene

## Abstract

**Background:**

Chronic rhinosinusitis (CRS) is a frequent disease with high social impact and multifactorial pathogenesis. Recently, single nucleotide polymorphisms within the *TAS2R38* gene have been implicated as possible contributors to the complex gene-environment interactions in CRS.

The purpose of this study was to confirm the proposed correlation between *TAS2R38* genotype, CRS and related comorbidities.

**Methods:**

Fifty-three CRS patients and 39 healthy individuals were genotyped at the *TAS2R38* locus. CRS patients were treated by endoscopic sinus surgery and medical therapies and subdivided in CRS with nasal polyps (CRSwNPs) and CRS without nasal polyps (CRSsNPs). The effect of genotype on CRS and CRS-related comorbidities was assessed.

**Results:**

The distribution of the different genotypes at the *TAS2R38* locus was not significantly different between CRS patients, either with or without nasal polyps, and controls. Besides, no association was found between the different genotypes at the *TAS2R38* locus and CRS-related comorbidities.

**Conclusions:**

No association was found between *TAS2R38* alleles or genotypes and CRS, thus questioning its role in the pathogenesis of CRS.

**Electronic supplementary material:**

The online version of this article (doi:10.1186/s12881-016-0321-3) contains supplementary material, which is available to authorized users.

## Background

Chronic rhinosinusitis (CRS) represents a burden on patients quality of life (QoL) [1, 2] and on healthcare system [3], as a result of a relevant prevalence in the general population (14–16 % in the United States [4], 10.9 % in Europe [5–7]).

Despite the remarkable efforts invested in the past decades, CRS pathophysiology is far from being completely elucidated. Currently, CRS is recognized as a multifactorial disease [8] arising from the contribution of different aetiological factors [9], although most of them are not definitively proven.

Recently, genetic polymorphisms of bitter taste receptors (T2Rs) have been proposed as contributors to CRS. T2Rs are metabotropic G protein coupled receptors (GPCR). When activated, they ultimately determine calcium release from intracellular deposits and consequent opening of TRPM5 (Transient Receptor Potential Channel M5), causing an action potential that triggers taste perception [10, 11]. In particular, T2R38, whose gene is located on chromosome 7q, is implicated in taste perception of phenylthiocarbamide (PTC), one of the genetic traits best characterized for its distribution in human populations [12]. Interestingly, T2Rs are expressed not only in gustatory cells but also in many other tissues [13, 14]. T2R38 expression has been found also in ciliated human sinonasal epithelial cells, where its function appears to be activated in vitro by acyl-homoserine lactones (AHLs) [15]. AHLs are quorum sensing molecules secreted by Gram-negative bacteria, such as *Pseudomonas aeruginosa*, involved in the regulation of gene expression to direct different bacterial life cycle processes, for instance biofilm production and pathogenicity [16, 17] Through calcium-dependent pathways, T2R38 increases nitric oxide (NO) production and mucociliary clearance (MCC), thus contributing to the mucosal innate immunity [18, 19]. The activity of the receptor depends on three common single nucleotide polymorphisms (SNPs) within the *TAS2R38* gene (A49P, V262A, I296P) [12, 18]. These SNPs tend to segregate together, yielding two common and some less frequent haplotypes. The two more common are the functional allele, characterised by the presence of proline, alanine and valine (PAV) at aminoacidic positions 49, 262 and 296, respectively [12] and the non-functional allele, with alanine, valine and isoleucine (AVI) at the same positions. The allelic distribution varies by geographic region and ethnicity, and has been reported to be 49 % PAV, 47 % AVI and 3 % AAV in populations of European descent [12]. The different aminoacidic changes in the T2R38 protein determine a different binding ability to its ligands and a diverse activation of the signal transduction cascade. However, it is not completely understood which specific polymorphic position (codons 49, 262 or 296) contributes most to the different aspects of T2R38 activity [20, 21]. The above-mentioned common haplotypes generate the 3 most common genotypes (PAV/PAV, PAV/AVI, AVI/AVI) whose phenotypes concern the degree of PTC taste perception (super-taster, intermediate-taster, non-taster) [12, 21–23].

Recent in vitro investigations demonstrated that the super-taster genotype (PAV/PAV) has a significantly increased response to pseudomonal quorum-sensing molecules compared with heterozygous (PAV/AVI) or homozygous non-taster (AVI/AVI) genotypes, resulting in an increase in both NO production and ciliary beat frequency [18].

The hypothesis that a PAV/PAV genotype would be therefore protective against Gram-negative sinonasal infections and related chronic inflammation, was tested in a small pilot-study by Adappa and collaborators, that showed a statistically significant paucity of PAV/PAV compared to the expected distribution (*p* < 0.043) in a cohort of CRS patients undergoing primary functional endoscopic sinus surgery (FESS) after failure of medical treatment [17]. This result was confirmed by the same authors in a larger study, [24] and recently refined, showing that the TAS2R38 genotype predicts the surgical outcome in nonpolypoid chronic rhinosinusitis [25].

Based on these promising findings, we wished to: [1] test whether the different genotypes at the *TAS2R38* locus were differentially represented within a prospectively selected Italian population of CRS and [2] investigate the possible correlation between *TAS2R38* genotype and CRS related factors (comorbidities, bacterial infection, number of surgeries).

## Methods

### Study

A prospective case-control study was carried out on a total of 92 individuals, comprising 53 cases (CRS) and 39 controls (CTL).

#### Cases (CRS)

We recruited patients affected by CRS with nasal polyps (CRSwNP) or without nasal polyps (CRSsNP), as defined by the European guidelines [6], referring to the outpatient clinic for routine follow-up. We included any 18 to 75 year-old patient with a history of at least one previous endoscopic sinus surgery performed in our tertiary care hospital, after medical treatment failure, with a minimum 6 months follow-up from the last surgical procedure.

We excluded any patients belonging to the V class of Stammberger classification for CRSwNP [26], those affected by primary and secondary immunodeficiency or with history of sinonasal trauma and loco-regional radiotherapy.

Information concerning age, sex, ethnicity, phenotype of CRS, age at first surgery, total number of surgical procedures, smoke habit, allergies, asthma, ASA sensitivity, nasal microbiological data and family history of CRS was collected for the whole cohort. Finally, venous blood samples were collected from each patient for *TAS2R38* genotyping.

#### Controls (CTL)

We recruited healthy volunteers, matched to patients for age and sex, among the healthcare staff of our institute about to undergo routinely hospital employment blood examination. The same demographic and selected clinical data, as for the cases cohort, were collected. Exclusion criteria consisted of a positive history of asthma, allergy and acute or recurrent rhinosinusitis. All controls underwent venous blood collection for *TAS2R38* genotyping purpose. We adjusted the controls selection for geographic region and ethnicity in order to minimize the environmental differences with the cases.

### Ethics, consent and permissions

The Varese University Hospital Ethics Committee approved this study (Protocol approval n. MED/OTO/2013/1). All participants provided informed consent to use their samples for research purposes. Research was carried out in compliance with the Helsinki Declaration.

### DNA extraction

Five millilitres of whole blood were collected from each participant using Vacutainer tubes containing citric acid and dextrose as anticoagulant. Two hundred microliters of whole blood were used for DNA extraction with the Purelink Genomic DNA Kit (Life Technologies, Milan, Italy) following manufacturer instructions. Purified DNA was eluted in 100 μl of Tris-HCl 10 mM, pH 8.0–9.0 and stored at 4 °C. DNA quality and concentration were checked by a Nanodrop 2000c spectrophotometer (Thermoscientific, Milan, Italy) and by agarose gel electrophoresis. For genotyping, DNAs were diluted in Polymerase Chain Reaction (PCR) grade water at 10 ng/μl and checked again by agarose gel electrophoresis to ensure that equal amounts were used in the analysis.

### Genotyping

The genotype at the *TAS2R38* locus was determined by allelic discrimination using the TaqMan 5’-nuclease assays (TaqMan SNP Genotyping Assays; Applied Biosystems, Milan, Italy), on a CFX96 platform (Biorad, Milan, Italy). The following SNPs were characterized: rs713598, rs1726866 and rs10246939, involving codons 49, 262 and 296, respectively.

Each PCR reaction was performed in 20 μl and contained 10 μl of TaqMan Universal PCR master mix, 1 μl of TaqMan genotyping assay (pre-diluted to 20x), 1 μl of genomic DNA diluted at 10 ng/μl and PCR grade water to volume. Two “No Template Controls” (NTC) were prepared for each SNP in each plate, to check for absence of contamination and to orient the graph for analysis. The amplification program performed an initial denaturation at 95 °C for 10’, followed by forty cycles of denaturation at 95 °C for 15” and annealing/extension at 60 °C for 1’. The results of the genotyping were analysed with the Biorad CFX manager software, version 3.1, and also manually inspected.

The genotype was scored for each subject.

### Statistical analysis

The allele frequencies were determined by direct gene count method. The observed genotype frequencies were tested for Hardy-Weinberg equilibrium using the *χ*^2^ test, after applying the Yates correction due to the presence of rare genotypes. The *χ*^2^ test with the Yates correction was also used to verify the null hypothesis that the likelihood of having CRS depends on the genotype at *TAS2R38* locus, by comparing the differences in allele frequencies between healthy and diseased individuals. Statistical analysis was performed using R [27].

## Results

Ninety-two individuals were enrolled in the present study. Thirty-nine were healthy controls and 53 were patients affected by CRS, subdivided in 36 cases with nasal polyps (CRSwNP) and 17 without nasal polyps (CRSsNP).

Upon genotyping, only 6 genotypes were observed in the whole cohort, derived by the combination of the two most frequent PAV, AVI alleles and the relatively frequent AAV allele (Table 3). Genotypes derived by other combinations of rarer alleles were not found in our analysis. Relevant epidemiological and clinical data as well as the observed genotypes distribution in CRS and CTL are reported in Table 1.

**Table 1:** Demographics and clinical data of the examined population

	CTL	CRS
Gender, n (male; female)	39 (20; 19)	53 (27; 26)
Age, years (mean ± SD)	46.6 ± 11.9	52.8 ± 14.7
Ethnic group, n (%)		
• Caucasian	38 (97.4)	52 (98.1)
• North African	1 (2.6)	1 (1.9)
CRS phenotype		
• CRSwNP, n (%)	-	36 (67.9)
• CRSsNP, n (%)	-	17 (32.1)
Age at first sinus surgery, years (mean ± SD)	-	42.9 ± 15.6
Endoscopic sinus surgeries under general anesthesia (mean ± SD)	-	2.4 ± 1.7
History of asthma, n (%)	-	30 (56.6)
History of allergy, n (%)	-	29 (54.7)
History of ASA sensitivity, n (%)	-	12 (22.6)
Smoking (current smoker/ex-smoker/non smoker), n (%)	10 (25.6)/6 (15.4)/23 (59)	1 (1.9)/23 (43.4)/29 (54.7)
Family history of CRS/asthma/allergy, n (%)	14 (35.9)	23 (43.4)
TAS2R38 genotype		
• PAV/PAV, n (%)	7 (17.9)	8 (15.1)
• PAV/AVI, n (%)	17 (43.6)	29 (54.7)
• AVI/AVI, n (%)	12 (30.8)	10 (18.9)
• PAV/AAV, n (%)	1 (2.6)	3 (5.7)
• AVI/AAV, n (%)	2 (5.1)	2 (3.8)
• AAV/AAV, n (%)	-	1 (1.9)

SD: standard deviation; CRSwNP: chronic rhinosinusitis with nasal polyps; CRSsNP: chronic rhinosinusitis without nasal polyps; ASA: acetyl-salicylic acid

Calculation of the expected genotypic frequencies from the allelic frequencies of the sample allowed us to demonstrate that our sample population is at the Hardy-Weinberg equilibrium (*χ*^2^_[4]_ = 3.36; *p* = 0.499).

Considering the presumed protective role of the PAV allele towards chronic airway inflammation and infection, further analyses were performed by clustering *TAS2R38* genotype according to a dosage effect of the PAV allele, that is the presence of two (PAV/PAV), one (PAV/-) and no copies (−/−).

A *χ*^2^ test of independence was then applied, in order to investigate whether a particular genotype was related to CRS phenotype. For this test we subdivided the CRS patients into those with and without nasal polyps (CRSwNP and CRSsNP, respectively). No difference in genotypic distribution was found (*χ*^2^_[4]_ = 2.323, *p* = 0.677), strongly suggesting a lack of association between any observed genotype and CRS (Table 2).

**Table 2:** Observed distribution of genotypes at the *TAS2R38* locus in the different categories of our cohort, used for association analysis

	CTL	CRS		Tot.
		CRSwNP	CRSsNP	
PAV/PAV	7	6	2	15
PAV/-	18	22	10	50
−/−	14	8	5	27
Tot.	39	36	17	92

The number of subjects presenting a particular genotype in each category (CTL, CRS, CRSwNP, CRSsNP) was multiplied by Π in order to calculate the expected genotypic frequencies

Π represents the fraction of individuals falling in each category on the total of subjects [Π_(CTL)_ = 0.424 (39/92); Π_(CRSwNP)_ (36/92) = 0.391; Π_(CRSsNP)_ (17/92) = 0.185]

Association between genotypes at the *TAS2R38* locus and CRS was tested by *χ* ^2^

Association between genotype at the *TAS2R38* locus and other CRS related factors was analysed as well, as reported in Table 3. Again, no significant association was found (asthma: *χ*^2^_[2]_ = 1.682; *p* = 0.431; allergy: *χ*^2^_[2]_ = 1.241; *p* = 0.538; ASA sensitivity: *χ*^2^_[2]_ = 2.234; *p* = 0.327; number of surgeries: *χ*^2^_[2]_ = 1.030; *p* = 0.598).

**Table 3:** Observed distribution of genotypes at the *TAS2R38* locus and CRS comorbidities (asthma, allergy, ASA sensitivity) or number of endoscopic surgical procedures in CRS patients, used for association analysis

	Asthma		Allergy		ASA sensitivity		N. surgeries		Tot.
	Present	Absent	Present	Absent	Present	Absent	1	>1	
PAV/PAV	6	2	3	5	2	6	4	4	8
PAV/-	18	14	19	13	9	23	10	22	32
−/−	6	7	7	6	1	12	5	8	13
Tot.	30	23	29	24	12	41	19	34	53

The following Π values were used to calculate the expected frequencies of each comorbidity (see Table 2): Π_(Absence of asthma)_ = 23/53 = 0.434; Π_(Absence of allergy)_ = 24/53 = 0.453; Π_(Absence of ASA sensitivity)_ = 41/53 = 0.774; Π_(1 surgery)_ = 19/53 = 0.358

Association between genotypes at the *TAS2R38* locus and CRS aggravating factors was then tested by *χ* ^2^

Given the proposed role of T2R38 in innate immune defence, we also tested whether a specific genotype was associated with an increased risk of sinonasal bacterial infection. However, no significant association was found (*χ*^2^_[3]_ = 0.998, *p* = 0.910) (Table 4).

**Table 4:** Observed distribution of genotypes at the TAS2R38 locus and presence and type of bacterial infection within the CRS patients, used for association analysis

	Bacterial infection			
	Absent	Present		Tot.
		Gram +	Gram – and mixed flora	
PAV/PAV	4	2	2	8
PAV/-	12	6	14	32
−/−	5	3	5	13
Tot.	21	11	21	53

The following Π values were used to calculate the expected frequencies of each comorbidity (see Table 2): Π_(Absence of bacterial infection)_ = 21/53 = 0.396; Π_(Presence of Gram +)_ = 11/53 = 0.453; Π_(Presence of Gram – and mixed flora)_ = 21/53 = 0.396

Association between genotypes at the *TAS2R38* locus and bacterial infection was then tested by *χ* ^2^

## Discussion

A genetic basis for CRS has been postulated long ago. Several pieces of evidence strongly suggest the existence of a genetic component in CRS, for example the association between nasal polyposis and cystic fibrosis, a familiar heritability up to 42 % [28] and a concordance in the development of sinusitis in identical twins. This prompted the search for genes contributing to CRS susceptibility. A large majority of studies focused on the identification of polymorphisms in genes related to innate immune defence or in regulatory elements of CRS inflammatory cascade.

However, despite a conspicuous amount of literature published, the genetic susceptibility factors to CRS remains largely unknown.

Recently, the bitter taste receptor T2R38, expressed on human sinonasal ciliated epithelial cells, has been proposed to activate in vitro mucosal innate immune defences, by acting as a detector of Gram-negative quorum sensing molecules (AHLs) and consequently activating calcium-dependent pathways leading to an increased NO production and MCC, to protect against airborne infections [18]. Similarly, T2R38 was shown to be the long sought receptor for the Gram negative quorum sensing molecule AHL-12 in neutrophils [14].

T2R38 receptor activity depends on the status at three common polymorphisms within the *TAS2R38* gene. The functional allele, in its homozygous state (PAV/PAV), has been proposed to exert a protective effect in response to bacterially secreted AHLs, whereas the homozygous AVI/AVI genotype would not defend against bacterial infections [18].

The first pilot-study by Adappa et al. was conducted on 28 patients, undergoing primary FESS, who were genotyped for *TAS2R38* and followed-up for 6 months after surgery [17]. Adappa et al. found that 33 % of the PAV/AVI genotype patients and 44 % of the AVI/AVI genotype patients required at least 1 additional course of postoperative antibiotics (interpreted as a persistence or a relapse of disease), whereas the single patient with PAV/PAV genotype within the cohort had favourable outcomes. A significant difference in genotype frequencies was found between their cohort and other populations described in the literature, although no conclusions were drawn, given the small number of cases examined. In a second paper by the same authors [24], a larger group of patients (70, including the previously reported cases) was analysed in comparison to a control population extracted from a research investigation on taste and smell by Mennella et al. [23]. Results confirmed their previous observations on a higher frequency of AVI/AVI genotype in the medically refractory CRS population than in the comparison sample. Recently, the same authors refined the prognostic value of the PAV/PAV genotype which appears to predict a favourable outcome after surgery (in term of QoL) in CRS patients without nasal polyps [25].

Since T2R38 activity can be measured in a semi-quantitative manner using a taste test with PTC strips [29], evidence of an involvement of specific *TAS2R38* genotypes in CRS susceptibility or prognosis (favourable versus poor outcomes) could be exploited to easily identify patients at risk of difficult-to-treat-sinusitis, without expensive genotyping tests. All these promising assumptions were the reason for the present prospective study.

Genotyping of our cohort of 92 subjects revealed a genotype distribution comparable to the distribution reported in other larger series published in the literature [23]. Our genotyping was made for each of the three SNP separately. Thus, if a subject was heterozygous at two or three of the SNPs, alternative genotypes might be possible. However, since other eventual genotypes would be combinations of rare or extremely rare haplotypes, and rare alleles were never found in our cohort even in combination with common ones, heterozygous genotypes were inferred to be PAV/AVI. The only way to genotype without uncertainties would be to clone and sequence the single alleles of the subjects carrying heterozygous genotypes, but this is not commonly performed for any such study [12, 23].

In our study no significant difference in the distribution of the three more common genotypes (PAV/PAV, PAV/AVI, AVI/AVI) did emerge by comparing CRS patients and healthy controls.

It is true that our cohort shows an overall high prevalence of nasal polyps (67.9 %), asthma (56.6 %) and allergy (54.7 %). These clinical aspects often concur with a CRS phenotype known to be closely associated to eosinophil levels and Th2 cytokine skewing, with higher risk for refractory disease [6]. However, we found neither significant differences in genotype distribution between CRSwNP and CRSsNP (Table 2), nor significant association between any CRS related factors and a particular genotype (Tables 3 and 4). Finally, the demographic and clinical features of the present cohort do not substantially differ from those deduced by previous reports (Additional file 1: Table S1), in which *TAS2R38* genotype seemed to play a role in determining CRS clinical outcomes [24, 25].

This different behaviour can be explained based on the complexity of the trait studied, which is not probably under the control of a single genetic factor. The different genetic makeup of the populations studied in our work and in those by Adappa and colleagues is therefore to be considered when drawing inferences about TAS2R38 and rhinosinusitis. The present work is undoubtedly not exempt from limitations, first of all due to the relatively small number of individuals included. In light of these findings, further studies that include many more patients collected in a prospective manner with long-term follow-up are necessary to establish a role for *TAS2R38* genotype in CRS pathogenesis and clinical decision-making.

Only one of the *TAS2R38* SNPs, namely rs10246939 (I296V), was found to be weakly associated to CRS in a previous pooling-based genome-wide association study (pGWAS), with an allele frequency difference in cases compared to controls ranging between 11.1 and 15.1 % in two different populations [30]. However, allelotyping, deriving from a pGWAS, does not provide information about haplotype frequencies and requires individual genotyping to counter errors occurring during the estimation from DNA pools.

Furthermore, it is still unclear how heterozygous individuals (PAV/AVI), who are the most represented in our and other series [23, 24], should be considered regarding a potential susceptibility to CRS. This is the reason why we preferred to maintain 3 genotypic classes (PAV/PAV, PAV/-, −/−) in our analysis. It has to be borne in mind that the ancestral role of this gene is probably related to food choice and protection from ingesting toxic substances, present in some vegetables, rather than defence against airways infections, since variation in the bitter taste perception predates the divergence between humans and Neanderthals. In fact, a heterozygous P/A (probably PAV/AVI) Neanderthal individual has been found [31]. The maintenance of different alleles thus appears to be an effect of balancing selection at the *TAS2R38* locus. In vitro, T2R38 activation following AHLs exposure appears to be absent in both AVI/AVI homozygous and PAV/AVI heterozygous cells [18], although response to the classical ligand, PTC, highlights a difference between AVI/AVI and PAV/AVI individuals: the latter can taste PTC strips albeit with high inter-individual variability.

Additionally, it should not be forgotten that T2R38 activity has been tested only in vitro and with Gram-negative bacteria molecules (AHLs), although the microbial community of CRS is known to include a much wider range of bacterial and fungal species [32].

## Conclusions

In conclusion, in the present study we did not find an association between *TAS2R38* genotype and CRS, thus questioning its real contribution to CRS susceptibility. It is also fair to say that, even if no association was found, we cannot rule out that there might be downstream mechanisms, regarding for example gene expression regulation, which may positively or negatively influence T2R38 functionality. Further studies on larger cohorts are needed to verify these findings also in vivo and to shed light on the role of bitter taste receptors in CRS.

## Abbreviations

CRS, Chronic rhinosinusitis; QoL, quality of life; TRPM5, Transient Receptor Potential Channel M5; GPCR, G protein coupled receptors; T2R, bitter taste receptor; PTC, phenylthiocarbamide; AHLs, acyl-homoserine lactones; NO, nitric oxide; MCC, mucociliary clearance; SNPs, single nucleotide polymorphism; FESS, functional endoscopic sinus surgery; CRSwNP, CRS with nasal polyps; CRSsNP, CRS without nasal polyps; CTL, Controls

## Additional file

Additional file 1: Table S1. Comparison of demographic and clinical data of patients analysed in the present study and two previous reports by Adappa et al. [24, 25]. (DOCX 22 kb)
