# Supplementary material for: TAS2R38 taste receptor gene and chronic rhinosinusitis: new data from an Italian population
Source: BMC Med Genet. 2016 Aug 11;17:54. doi: 10.1186/s12881-016-0321-3 (PMC4982233; doi:10.1186/s12881-016-0321-3)
Supplement: Additional file 1: Table S1. — Comparison of demographic and clinical data of patients analysed in the present study and two previous reports by Adappa et al. [24, 25]. (DOCX 22 kb) [file 12881_2016_321_MOESM1_ESM.docx]

|  | Present study  non PAV/PAV  (n=45) | Present study    PAV/PAV  (n=8) | Adappa et al. (2014)  PAV/AVI or AVI/AVI  (n=64) | Adappa et al. (2014)  PAV  (n=6) | Adappa et al. (2016)  PAV/AVI or AVI/AVI  (n=167) | Adappa et al. (2016)  PAV  (n=40) |
| --- | --- | --- | --- | --- | --- | --- |
| Gender |  |  |  |  |  |  |
| - Male, n (%) | 23 (51.1) | 4 (50) | 43 (67) | 5 (83) | 98 (59) | 22 (55) |
| - Female, n (%) | 22 (48.9) | 4 (50) | 21 (33) | 1 (17) | 69 (41) | 18 (45) |
| Age at first surgery  (years ± SD) | 41.9 ± 15.4 | 46.6 ± 16.6 | - | - | 49±13 | 51±12 |
| Ethnicity |  |  |  |  |  |  |
| - Caucasian | 45 (100) | 7 (87.5) |  |  | 151 (90) | 33 (83) |
| - North african | - | 1 (12.5) |  |  | - | - |
| Clinical characteristics |  |  |  |  |  |  |
| - Current or ex smoker, n (%) | 19 (42.2) | 5 (62.5) | 5 (7.8) | 2 (33.3) | 59 (37) | 14 (37) |
| - Polyps, n (%) | 30 (66.7) | 6 (75) | 36 (56.3) | 2 (33.3) | 112 (67) | 30 (75) |
| - Asthma, n (%) | 24 (53.3) | 6 (75) | 26 (40.6) | 1 (16.7) | 90 (54) | 25 (63) |
| - Allergy, n (%) | 26 (57.8) | 3 (37.5) | 37 (57.8) | 3 (50) | 113 (67) | 26 (65) |
| - ASA sensitivity, n (%) | 10 (22.2) | 2 (25) | 2 (3.1) | 0 | 21 (13) | 11 (28) |
| Mean number of surgeries, (mean ± SD) | 2.6 ± 1.8 | 1.5 ± 0.5 | - | - | - | - |

**Additional Table 1**. Comparison of demographic and clinical data of patients analysed in the present study and two previous reports by Adappa et al.*

* References [24] [25], see text**.**
